# Performance Comparison of Diffusion Kurtosis Imaging (DKI), Neurite Orientation Dispersion and Density Imaging (NODDI), and Diffusion Microstructure Imaging (DMI) in Predicting Adult-Type Glioma Subtype—A Pilot Study

**DOI:** 10.3390/cancers17050876

**Published:** 2025-03-03

**Authors:** Leonie Zerweck, Urs Würtemberger, Uwe Klose, Marco Reisert, Vivien Richter, Thomas Nägele, Deborah Staber, Tong Han, Mi Shen, Chuanmiao Xie, Hongjie Hu, Songlin Yang, Zhijian Cao, Gunter Erb, Ulrike Ernemann, Till-Karsten Hauser

**Affiliations:** 1 Department of Diagnostic and Interventional Neuroradiology, University Hospital Tuebingen, 72076 Tuebingen, Germanytill-karsten.hauser@med.uni-tuebingen.de (T.-K.H.); 2 Department of Neuroradiology, Medical Center, Faculty of Medicine, University of Freiburg, 79106 Freiburg im Breisgau, Germany; 3 Department of Radiology, Tianjin Huanhu Hospital, Tianjin 300350, China; 4 Department of Radiology, Beijing Tian Tan Hospital, Capital Medical University, Beijing 100070, China; 5 Department of Medical Imaging, Sun Yat-sen University Cancer Center, Guangzhou 510060, China; 6 Department of Radiology, Sir Run Run Shaw Hospital, School of Medicine, Zhejiang University, Hangzhou 310018, China; 7 Department of Radiology, The Fifth Affiliated Hospital of Sun Yat-sen University, Zhuhai 519082, China; 8 Department of Radiology, The First Affiliated Hospital of Zhejiang Chinese Medical University, Hangzhou 310053, China; 9 Bracco Group, Medical and Regulatory Affairs, 78467 Konstanz, Germany

**Keywords:** glioma, glioblastoma, astrocytoma, oligodendroglioma, diffusion kurtosis imaging, neurite orientation dispersion and density imaging, diffusion microstructure imaging

## Abstract

**Background/Objectives**: The purpose of this study was to evaluate the performance of diffusion kurtosis imaging (DKI), neurite orientation dispersion and density imaging (NODDI), and diffusion microstructure imaging (DMI) in differentiating molecular subtypes of adult-type gliomas. **Methods**: Standardized MRI was performed and evaluated in 59 patients with adult-type glioma. DKI, NODDI, and DMI parameter values were quantitatively evaluated in ROIs in contrast-enhancing/solid tumor tissue and five concentric shells with peritumoral tissue. DKI, NODDI, and DMI parameters of (i) glioblastomas, Isocitrate dehydrogenase (IDH) wildtype; (ii) astrocytomas, IDH mutant; and (iii) oligodendrogliomas, IDH mutant were compared with analysis of variance (ANOVA). Receiver operating characteristic curve (ROC) curve analysis was conducted to discriminate firstly between IDH mutant and IDH wildtype gliomas and then between IDH mutant astrocytomas and oligodendrogliomas. **Results**: Significant differences between the three aforementioned subtypes were found for the apparent diffusion coefficient (ADC) and mean kurtosis (MK) and again for the orientation dispersion index (ODI) and intra-axonal volume fraction (v-intra). The diagnostic accuracy depended on the distance to the contrast-enhancing/solid tumor tissue. Some NODDI and DMI parameters significantly predicted the IDH status and significantly discriminated between astrocytomas and oligodendrogliomas; however, ADC and MK showed the best prediction in both ROC analyses (maximum AUC 0.910 (CI 0.824–0.995)). **Conclusions**: The evaluation of peritumoral tissue can be a valuable procedure, while NODDI and DMI appear to be promising but are currently inferior to DKI in predicting glioma subtypes categorized according to the WHO 2021 classification.

## Simple Summary

Diffusion-weighted imaging (DWI) is commonly integrated in magnetic resonance imaging (MRI) protocols when characterizing gliomas. Novel multicompartment diffusion MRI models, such as neurite orientation dispersion and density imaging (NODDI) as well as diffusion microstructure imaging (DMI), can provide microstructural information and seem to be useful in oncological neuroimaging. Therefore, in this pilot study, we aimed to explore the diagnostic performance of DKI, NODDI, and DMI in differentiating molecular subtypes of adult-type diffuse gliomas categorized by the World Health Organization (WHO) 2021 classification. Our results indicate that DKI appears to be superior to multicompartment diffusion MRI and that the evaluation of peritumoral tissue warrants attention.

## 1. Introduction

Identifying gliomas and furthermore distinguishing their subtypes, based on the World Health Organization (WHO) 2021 classification of central nervous system (CNS) tumors, is critical for assessing patient prognosis and selecting the most effective clinical management [1]. Tumor subtyping depends on histopathological and molecular analysis obtained through stereotactic biopsy or surgical resection [2,3]. Nonetheless, a reliable, non-invasive approach for tumor evaluation is needed in the primary diagnosis to determine the volume of tumor resection that extends beyond the primary contrast-enhancing tissue [4,5] and in follow-up or for monitoring for potential tumor recurrence [6].

Magnetic resonance imaging (MRI) is the imaging modality of choice for primary diagnosis and follow-up [7,8]. Additionally, diffusion-weighted imaging (DWI) can be included in the MRI imaging protocol when characterizing gliomas [9]. Standard DWI assumes that the water molecule diffusion follows a Gaussian distribution [3,10,11], yet in complex biological tissues, the presence of cell membranes and water compartments results in non-Gaussian diffusion behavior [3,10,11]. Diffusion kurtosis imaging (DKI) is an advanced diffusion imaging technique that does not rely on the assumption of Gaussian water diffusion but instead quantifies deviations from Gaussian behavior [3,6,11,12]. By providing additional microstructural insights, DKI seems to enable improved glioma grading compared to traditional diffusion parameters and morphological imaging [6,13,14].

Novel multicompartment diffusion MRI models, such as neurite orientation dispersion and density imaging (NODDI) and diffusion microstructure imaging (DMI), can provide microstructural information and seem to be useful in oncological neuroimaging [10,15,16,17,18,19,20]. These two approaches employ a three-compartment biophysical model consisting of three microstructural components within a single voxel: the intra-neurite fraction (within axons and dendrites) with highly restricted diffusion perpendicular to neurites and unhindered diffusion along them; the extra-neurite fraction containing glial cells, neuronal cell bodies, and extracellular space with hindered diffusion by the presence of neurites; and the cerebral spinal fluid (CSF) fraction with unrestricted isotropic diffusion [19,21,22,23,24].

NODDI uses the parameters intracellular volume fraction (ficvf), the orientation dispersion index (ODI), and the isotropic volume fraction (fiso) to characterize the microstructure within a voxel [21] and assumes that the diffusion within each compartment follows Gaussian behavior [21]. The ficvf quantifies the intra-neurite volume fraction [24], the ODI assesses the orientation dispersion of the neurites [24], while the fiso estimates the amount of free water/CSF with isotropic diffusion in a voxel [21].

DMI represents a further development using a Bayesian estimator to model the microstructural tissue properties [18,20,25] relatively quantifying the following volume compartments: the intra-axonal volume fraction (v-intra), the extra-axonal volume fraction (v-extra), and the free water/CSF fraction (v-CSF) [18,20].

The purpose of this study was to quantitatively compare the diagnostic performance of DKI, NODDI, and DMI parameters in molecular subtype identification according to the WHO 2021 classification of CNS tumors, which divides adult-type gliomas into (i) glioblastomas, Isocitrate dehydrogenase (IDH) wildtype; (ii) astrocytomas, IDH mutant; and (iii) oligodendrogliomas, IDH mutant and 1p/19q-codeleted [2,26,27].

To our knowledge, there are currently no studies that have compared the different DWI techniques DKI, NODDI, and DMI in the diagnosis of brain tumors according to the WHO 2021 classification. Therefore, in this study, we decided to focus on a comparison of the three DWI approaches, especially in different regions, and excluded other advanced MRI techniques such as perfusion-weighted imaging (PWI), magnet resonance spectroscopy (MRS), and chemical exchange saturation transfer (CEST) imaging.

## 2. Materials and Methods

### 2.1. Study Design

A prospective study was conducted in six neurosurgical centers in China. The study adhered to the principles of the Declaration of Helsinki and was approved by the local ethics committee at each center. Written informed consent was obtained from all participants.

### 2.2. Participants

A total of 108 patients with suspected adult-type cerebral glioma were recruited and underwent imaging using a standardized MRI protocol. Inclusion criteria were the presence of a suspected supratentorial adult-type glioma and a planned cerebral tumor biopsy and/or surgery, with complete histopathologic analysis according to the most recent WHO 2021 classification of CNS tumors [2] performed within four weeks following the study MRI. Patients were excluded if they had contraindications to MRI (e.g., pacemakers, metal implants, pregnancy, allergy to contrast agents, severe renal impairment with GFR/eGFR < 30 mL/min, severe claustrophobia, etc.) and if they had received radiotherapy or chemotherapy prior to the biopsy or surgery.

### 2.3. MR Imaging

All patients underwent imaging with a 3T MRI scanner. All devices were Siemens-manufactured to ensure consistent image quality and comparability across all study sites.

The imaging protocol involved conventional MRI sequences, including axial T1 spin echo (SE)/fast spin echo (FSE) sequences before and after contrast administration (Gadobenate dimeglumine (MultiHance; Bracco, Milano, Italy) using 0.1 mmol per kg body weight), axial T2 FLAIR, and post-contrast 3D-T1 gradient echo (GRE) imaging and DWI. The DWI images for DKI, NODDI, and DMI analysis were conducted using a 2D echo-planar imaging (EPI) sequence with multiple b-values (0, 500, 1000, 1500, 2000, 2500 s/mm^2^) for diffusion analysis (TR 5900 ms, TE 95 ms, voxel size 2.0 × 2.0 × 5.0 mm, EPI factor 128).

### 2.4. Image Analysis

The post-processing and analysis of the MRI data were performed off-site in Germany.

The values of the parameters, apparent diffusion coefficient (ADC), and mean kurtosis (MK), were calculated using scripts written in MATLAB (R2018b (The MathWorks, Inc., Natick, MA, USA)). NODDI MATLAB toolbox was used to calculate the values of the NODDI-derived parameters ODI, ficvf, and fiso. After preprocessing, including a denoising step [28], correction of Gibbs-ringing artifacts [29], and up-sampling to isotropic resolution, the DMI parameter values were calculated with a Bayesian approach [25], as recently described [15,16,17,18].

The regions of interest (ROI) were defined using the anatomical non-contrast T1, post-contrast T1, FLAIR, and T2 images. The ROIs of the DWI images were obtained after registration of the DWI images and the anatomical non-contrast T1, post-contrast T1, FLAIR, and T2 images.

The medical open network for artificial intelligence (MONAI) framework (2024)., an open-source deep learning toolkit, with a brain tumor segmentation (BraTS) model, was utilized for image segmentation [30]. BraTS models are deep learning models that were developed as part of the BraTS challenge, an initiative to improve automated MRI segmentation [31]. For contrast-enhancing tumors, the BraTS glioma model was employed to segment the images into contrast-enhancing tumor, necrosis, and non-enhancing tumor/perifocal edema. For non-contrast-enhancing tumors, the BraTS model for pediatric tumors (BraTS-PED) was implemented. Tissue containing necrosis and cystic cavities were not included in the further analysis. CSF spaces and bones were systematically excluded.

**Figure 1:**
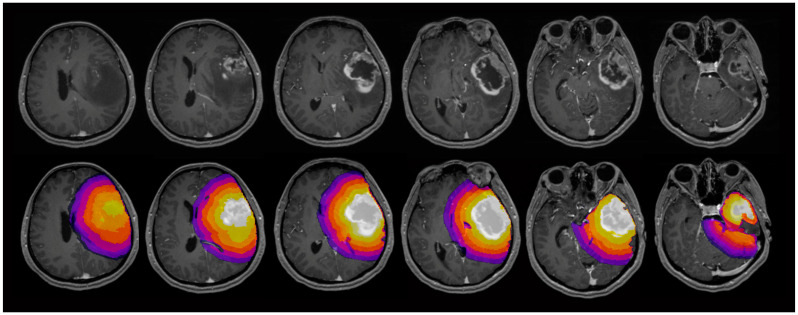
Exemplary maps of one patient with glioblastoma in the left temporal lobe (top row) and the evaluated regions of interest (ROI) (bottom row): The contrast-enhancing tumor was selected as ROI_1_. Five concentric shell-shaped ROIs (ROI_2–6_) with a distance of 5 mm between each ROI were defined. In total, 6 three-dimensional ROIs were evaluated.

**Figure 2:**
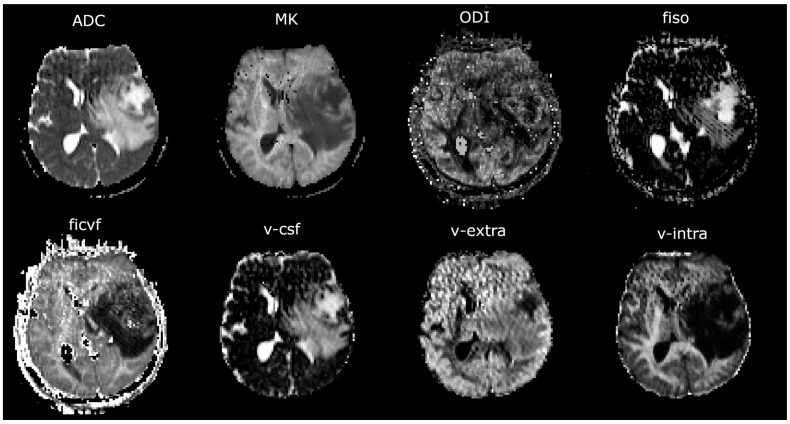
Exemplary maps of each image parameter of one patient with glioblastoma in the left hemisphere: ADC = apparent diffusion coefficient, MK = mean kurtosis, ODI = orientation dispersion index, fiso = isotropic volume fraction, ficvf = intracellular volume fraction, v-csf = free water fraction, v-extra = extra-axonal volume fraction, v-intra = intra-axonal volume fraction.

In total, 6 three-dimensional ROIs were analyzed. The contrast-enhancing tumor center or, in non-contrast-enhancing tumors, the solid tumor center was defined as ROI_1_. Five additional concentric ROIs (ROI_2_–ROI_6_) were defined in shells around the central ROI_1_, with a distance of 5 mm between each of the shells. Exemplary maps of the ROI selection can be seen in Figure 1, while Figure 2 illustrates exemplary maps of each evaluated parameter.

### 2.5. Postoperative Tumor Subtyping

Postoperative pathology reports were reviewed to determine the histopathologic diagnosis, tumor grade, and molecular tumor markers using the WHO 2021 classification of adult-type brain tumors [2]. We distinguished between [2,26]:

(i) glioblastomas, IDH wildtype
(ii) astrocytomas, IDH mutant
(iii) oligodendrogliomas, IDH mutant

IDH wildtype gliomas, not elsewhere classified (NEC), were not included in the analysis due to the expected small sample size.

### 2.6. Statistical Analysis

Statistical analyses were performed using SPSS Statistics (IBM Corp. Released 2021. IBM SPSS1 Statistics for Windows, Version 28.0. Armonk, NY, USA: IBM Corp). Statistical figures were generated with SPSS Statistics and MATLAB (R2018b (The MathWorks, Inc., Natick, MA, USA; http://www.mathworks.com)).

Normal distribution and homogeneity of variance of all DKI, NODDI, and DMI parameters were verified with the Shapiro–Wilk test and Levené test. Next, the means of each ROI were tested for group differences between (i) glioblastomas, IDH wildtype; (ii) astrocytomas, IDH mutant; and (iii) oligodendrogliomas, IDH mutant, using analysis of variance (ANOVA). Tukey post-hoc tests were performed.

Subsequently, the ability of all DKI, NODDI, and DMI parameters to differentiate between the groups (i)–(iii) was evaluated. For this purpose, univariate binary logistic regression analyses were performed to assess the diagnostic value of all parameters to differentiate between (a) IDH wildtype vs. IDH mutant gliomas and (b) astrocytomas vs. oligodendrogliomas. Receiver operating characteristic (ROC) curves were generated for each parameter to determine the area under the curve (AUC), sensitivity, and specificity. The results with the highest Youden index were defined as the optimal cutoff value. DeLong tests were performed to compare the AUC values of ADC, MK, and of the parameters of NODDI and DMI with the highest AUC.

The alpha level of each statistical test was *p* < 0.05, with false discovery rate (FDR) used to correct for multiple comparisons.

## 3. Results

### 3.1. Patients

A total of 108 patients with suspected cerebral glioma were enrolled. After histopathological review, 84 patients with adult-type supratentorial gliomas WHO grade 2–4 were included in the further analysis. Data sets with incomplete histopathologic/molecular results were excluded. Data sets of 25 patients were excluded due to insufficient image quality. Ultimately, 59 data sets were included. Patient characteristics are shown in Table 1.

### 3.2. Comparison of DKI, NODDI, and DMI Parameters in Each ROI

**Figure 3:**
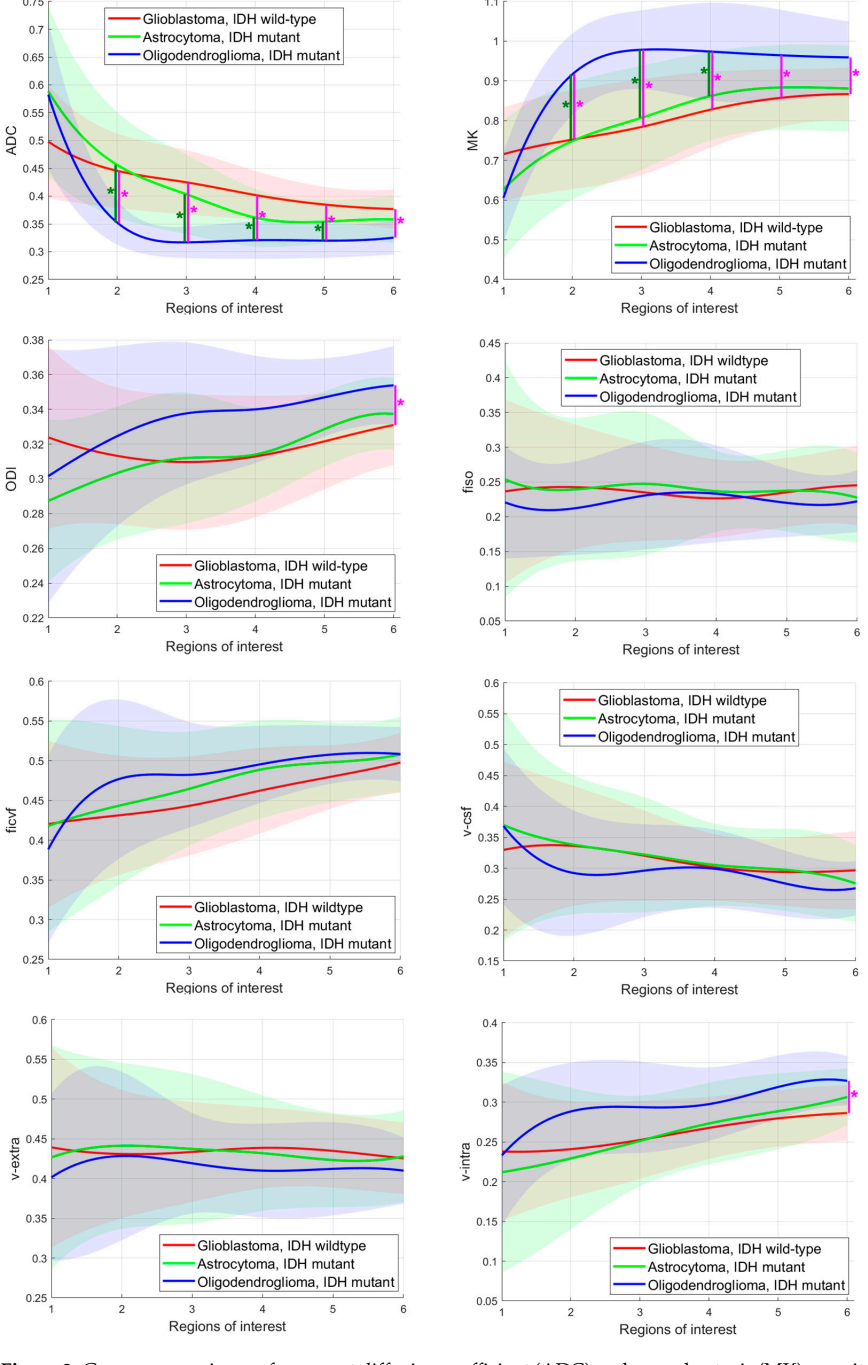
Group comparisons of apparent diffusion coefficient (ADC) and mean kurtosis (MK), neurite orientation dispersion and density imaging (NODDI) parameters orientation dispersion index (ODI), intracellular volume fraction (ficvf) and isotropic volume fraction (fiso) and diffusion microstructure imaging (DMI) parameters free water fraction (v-csf), extra-axonal volume fraction (v-extra) and intra-axonal volume fraction (v-intra). Values between the regions of interest are interpolated. The light bars show the mean values ± the standard deviation of each parameter. Significant differences are indicated by vertical lines between the corresponding graphs and by the asterisks (*).

Means and standard deviations of all glioma subgroups according to the ROI are shown in Figure 3. Significant differences between the three adult-type glioma subgroups were found for the parameters ADC in ROI_2_–ROI_6_, MK in ROI_2_–ROI_6_, ODI in ROI_6_, and v-intra in ROI_6._ For ADC, the post-hoc tests revealed significant differences between (i) glioblastomas, IDH wildtype and (iii) oligodendrogliomas, IDH mutant and in ROI_2_–ROI_6_ and significant differences between (ii) astrocytomas, IDH mutant and (iii) oligodendrogliomas, IDH mutant in ROI_2_–ROI_5_. For MK, the post-hoc tests showed significant differences between (i) glioblastomas, IDH wildtype and (iii) oligodendrogliomas, IDH mutant and in ROI_2_–ROI_6_ and additionally significant differences between (ii) astrocytomas, IDH mutant and (iii) oligodendrogliomas, IDH mutant in ROI_2–6_. ODI and v-intra revealed significant differences between (i) glioblastomas, IDH wildtype and (iii) oligodendrogliomas, IDH mutant in ROI_6_.

### 3.3. Diagnostic Performance in Differating IDH Mutant from IDH Wildtype Gliomas

**Figure 4:**
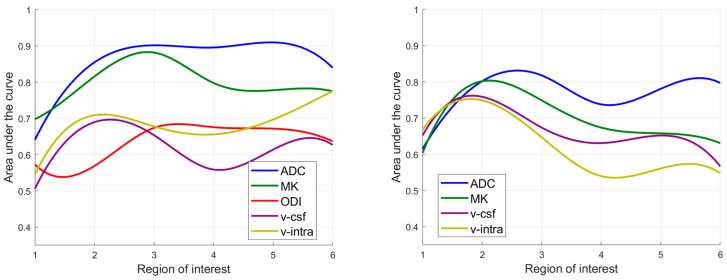
Selected area under the curve (AUC) values predicting the IDH status (**left**) and predicting astrocytomas vs. oligodendrogliomas (**right**) depending on the region of interest (ROI). ADC = apparent diffusion coefficient, MK = mean kurtosis, ODI = orientation dispersion index, v-csf = free water fraction, v-intra = intra-axonal volume fraction. The figure illustrates that the AUC of each parameter depends on the distance to the tumor center, e.g., the AUC of MK is highest in ROI_3_, while the AUC of v-intra is highest in ROI_6_.

ROC curve analysis revealed significant AUC values for the parameters ADC, MK, ODI, v-csf, and v-intra (see Table 2). To illustrate the dependence of the AUC value of each parameter on the distance from the tumor center, the AUC of the parameters with significant values was plotted against the ROI in Figure 4. Apart from ROI_1_, ADC showed the best diagnostic performance (maximum AUC of 0.910, confidence interval (CI) 0.824–0.995 in ROI_6_). In ROI_2–5_, the ADC showed significantly higher AUC values than the NODDI and DKI parameters with the best diagnostic performance (DeLong *p* = 0.044 vs. ficvf and *p* = 0.049 vs. v-intra in ROI_2_, *p* = 0.022 vs. ODI and *p* = 0.017 vs. v-intra in ROI_3_, *p* = 0.037 vs. ODI and *p* = 0.019 vs. v-intra in ROI_4_, *p* = 0.014 vs. ODI and *p* = 0.030 vs. v-intra in ROI_5_).

### 3.4. Diagnostic Performance in Differating Astocytomas, IDH Mutant from Oligodendrogliomas, IDH Mutant

Significant AUC values were found for the parameters ADC, MK, v-csf, and v-intra (see Table 3). Apart from ROI_1_, ADC showed the best diagnostic performance (maximum AUC of 0.802, CI 0.621–0.984 in ROI_3_), but the AUC was not significantly different from the AUC of the parameter of the other modalities with the best diagnostic performance. To show that the AUC value of each parameter depends on the distance to the tumor center, the AUC values of selected parameters with significant AUC values were plotted against the ROI in Figure 4.

## 4. Discussion

The aim of this preliminary study was to evaluate the performance of the diffusion MRI modalities DKI, NODDI, and DMI in molecular subtype identification according to the WHO 2021 classification of CNS tumors [2]. Based on histopathologic analysis performed within four weeks following the study MRI, we classified the gliomas into (i) glioblastomas, IDH wildtype; (ii) astrocytomas, IDH mutant; and (iii) oligodendrogliomas, IDH mutant [2]. NODDI and DMI are newly proposed diffusion MRI-based approaches that have shown promising results not only in the assessment of neurooncological lesions, but also in the perilesional tissue [16,18,20]. Therefore, we assessed not only contrast-enhancing and solid tumor regions, but also perilesional regions at different distances from the tumor center.

First, we investigated whether DKI, NODDI, and DMI parameters differed between the aforementioned glioma subgroups. ADC, MK, ODI, and v-intra revealed significant differences. The differences were mostly measurable between (i) glioblastomas, IDH wildtype and (iii) oligodendrogliomas, IDH mutant and 1p/19q codeleted and partly between (ii) astrocytomas, IDH mutant and (iii) oligodendrogliomas, IDH. Interestingly, significant differences were measured in the perilesional tissue, while no significant group differences were found in the contrast-enhancing or solid tumor center.

We then compared the diagnostic accuracy of all parameters to discriminate between the IDH status and between astrocytomas and oligodendrogliomas, as these binary comparisons allow classification into the three subgroups (i–iii). ADC, MK, ODI, v-csf, and c-intra significantly predicted the IDH mutation status, and ADC, MK, v-csf, and c-intra significantly discriminated between IDH mutant astrocytomas and oligodendrogliomas. ADC showed the best performance in both analyses, with a significantly higher AUC than the best parameter of the other modalities, NODDI and DMI, when analyzing the IDH mutation status. The diagnostic accuracy of the parameters depended on the distance to the contrast-enhancing or solid tumor tissue.

The IDH mutation status is one of the most important prognostic markers of gliomas [32]. Glioblastomas, IDH wildtype are more aggressive than IDH mutant astrocytomas and oligodendrogliomas and are expected to show a higher degree of cellularity, cell proliferation, angiogenesis, and microvascular density [19,32,33]. These factors could impede and restrict the diffusion of water molecules [19]. In our study, in the contrast-enhancing tissue of glioblastomas, IDH wildtype, tends towards lower ADC, higher MK, higher ODI, and high icvf and v-intra compared to IDH mutant gliomas were seen; however, the differences were not significant and should therefore be interpreted with caution. The observed tendency towards lower ADC, high icvf, and high v-intra values might be explained by the expected higher intracellular volume in glioblastomas, IDH wildtype. The tendency towards higher MK and higher ODI might be attributable to the expected more inhomogeneous cell architecture because ODI estimates the variability of fiber orientation [18] and MK also correlates with cellular heterogeneity [6]. Consistent with our study, high MK values in IDH wildtype gliomas have also been described by Hempel et al. [34], and the negative correlation between ADC and tumor cellularity has previously been demonstrated in the large meta-analysis of Chen et al. [35]. Interestingly, we observed that the parameters behaved inversely in the peritumoral edema (significantly higher ADC and significantly lower MK and lower v-intra in glioblastomas). Our results are consistent with those of Zhao et al. who also reported a tendency for higher ODI and higher icvf in the tumor parenchyma of IDH mutant gliomas and opposite behavior in peritumoral edema [19]. The parameter differences between the glioma subtypes in the peritumoral regions could be due to different proportions of vasogenic and cytotoxic edema and tumor infiltration, which may cause different diffusion behavior [20,36].

The distinction between astrocytomas and oligodendrocytomas is also clinically relevant, and not only because oligodendrocytomas are uniquely sensitive to chemotherapy [37,38]. Yang et al. described significantly lower ADC values in oligodendrogliomas, IDH mutant and 1p/19q-codeleted than in astrocytomas, IDH mutant and attributed this fact to the increased vascularity of oligodendrogliomas [39]. In this study, we detected comparable ADC values between astrocytomas and oligodendrogliomas in the solid tumor parenchyma, but significantly lower ADC values in the peritumor tissue of oligodendrogliomas. To the best of our knowledge, neither NODDI nor DMI has been evaluated for its ability to differentiate between astrocytomas and oligodendrogliomas.

Overall, in this study, ADC and MK demonstrated better performance in identifying glioma subtypes than the multicompartment diffusion MRI approaches. This not only aligns with Figini et al., who found no advantage of NODDI over diffusion tensor imaging in predicting the IDH status [40], but also with Zhao et al., who were unable to significantly predict the IDH status from NODDI data [19]. Nevertheless, promising results of microstructural diffusion MRI in differentiating between low-grade and high-grade gliomas [10,19,41], between glioblastomas and metastases [17,18,20], and between glioblastomas and lymphomas [16] have been described in the literature, warranting further research.

This study has several limitations. The main limitation was the limited sample size in certain subgroups due to the distribution of the included glioma subtypes according to their prevalence and the resulting relative rarity of astrocytomas, IDH mutant, whose differentiation from glioblastomas, IDH wildtype is relevant in clinical routine. For this reason, statistical statements should be interpreted with caution, and the study represents a pilot study that needs to be followed by further studies with larger sample sizes. The results of this pilot study must be validated and compared to established MRI examinations before they can be used clinically and before they have implications for surgical procedures. A potential future application involves utilizing preoperative subtype determination in patients who can only be operated on with increased risk of complication to weigh up further treatment options [34]. Another limitation was that metastatic brain tumors were not assessed and that the study focused on subtyping adult-type gliomas, rather than on glioma grading. In this study, we performed comparisons between the DWI modalities in standardized ROI, which enabled good comparability. Nonetheless, a limitation was that in the peripheral zones, which likely correspond to a mixed image of tumor infiltration, edema, and healthy tissue, no segmentation into grey and white matter was performed. This may have negatively affected the quality of the multicompartment approaches in NODDI and DMI. Furthermore, this study focused on diffusion MRI and did not combine other MRI modalities, as recommended by recent studies [32,39,42,43,44].

However, to the best of our knowledge, no study to date, has quantitatively compared the diagnostic performance of DKI, NODDI, and DMI in the identification of glioma subtypes based on the WHO 2021 classification of CNS tumors. Furthermore, only a few studies have evaluated DWI parameters in standardized peritumoral regions.

Further studies with larger sample sizes and multiparametric approaches are recommended to evaluate the diagnostic value of multicompartment diffusion MRI in glioma diagnostics.

## 5. Conclusions

This pilot study demonstrated that the evaluation of peritumoral tissue, which contains both non-contrast-enhancing tumor infiltration and edema, warrants attention. ADC and MK are useful parameters for noninvasive identification of adult-type glioma subtypes according to the WHO 2021 classification and appear to be superior to multicompartment diffusion MRI.

## Figures and Tables

**Table 1:** Patient characteristics.

Patients enrolled in the study	108
Patients included in the data analysis	59
Patients excluded due to histopathological/molecular diagnosis	24
Patients excluded due to insufficient MRI quality	25
Mean age of the included patients ± SD	45.3 ± 15.7
Female:male ratio	1:1.6
Glioblastoma, IDH wildtype (WHO grade 4)	31 (47.4%)
Astrocytoma, IDH mutant (WHO grade 2)	12 (18.5%)
Astrocytoma, IDH mutant (WHO grade 3)	1 (1.5%)
Astrocytoma, IDH mutant (WHO grade 4)	4 (6.2%)
Oligodendroglioma, IDH mutant (WHO grade 2)	3 (4.6%)
Oligodendroglioma, IDH mutant (WHO grade 3)	8 (12.3%)

**Table 2:** Diagnostic performance in differentiating IDH mutant from IDH wildtype gliomas of all diffusion kurtosis imaging (DKI), neurite orientation dispersion and density imaging (NODDI), and diffusion microstructure imaging (DMI) parameters in all regions of interest (ROI).

		ADC	MK	ODI	fiso	ficvf	v-csf	v-Extra	v-Intra
ROI_1_	AUC (95% Confidence interval)	0.641 (0.452–0.830)	0.698 (0.522–0.874)	0.573 (0.397–0.749)	0.547 (0.377–0.717)	0.525 (0.348–0.703)	0.506 (0.319–0.693)	0.549 (0.379–0.719)	0.545 (0.367–0.723)
	Cutoff value ^1^	0.536	0.561	0.638	0.164	0.624	0.283	0.416	0.619
	Sensitivity	0.933	0.867	0.667	0.767	0.767	0.700	0.733	0.767
	Specificity	0.529	0.588	0.588	0.412	0.412	0.412	0.412	0.353
ROI_2_	AUC (95% Confidence interval)	0.855 (0.738–0.972)	0.816 (0.672–0.959)	0.569 (0.395–0.742)	0.622 (0.458–0.785)	0.643 (0.477–0.810)	0.690 (0.531–0.849)	0.459 (0.284–0.633)	0.709 (0.555–0.863)
	Cutoff value ^1^	0.534	0.454	0.650	0.204	0.663	0.232	0.330	0.645
	Sensitivity	0.867	0.933	0.533	0.600	0.567	0.900	0.800	0.667
	Specificity	0.824	0.706	0.765	0.647	0.765	0.412	0.059	0.606
ROI_3_	AUC (95% Confidence interval)	0.902 (0.812–0.992)	0.882 (0.680–0.963)	0.674 (0.516–0.831)	0.558 (0.384–0.731)	0.655 (0.494–0.816)	0.654 (0.487–0.821)	0.529 (0.351–0.708)	0.678 (0.516–0.841)
	Cutoff value ^1^	0.723	0.485	0.664	0.191	0.601	0.304	0.439	0.652
	Sensitivity	0.767	0.993	0.633	0.800	0.700	0.633	0.633	0.600
	Specificity	0.941	0.665	0.765	0.412	0.588	0.765	0.647	0.606
ROI_4_	AUC (95% Confidence interval)	0.894 (0.797–0.991)	0.798 (0.638–0.958)	0.676 (0.520–0.833)	0.504 (0.328–0.680)	0.642 (0.481–0.803)	0.559 (0.387–0.731)	0.564 (0.387–0.741)	0.656 (0.489–0.823)
	Cutoff value ^1^	0.737	0.627	0.690	0.226	0.668	0.262	0.429	0.626
	Sensitivity	0.833	0.800	0.533	0.367	0.533	0.767	0.633	0.633
	Specificity	0.882	0.824	0.824	0.471	0.824	0.412	0.647	0.647
ROI_5_	AUC (95% Confidence interval)	0.910 (0.824–0.995)	0.778 (0.609–0.948)	0.672 (0.513–0.830)	0.594 (0.428–0.760)	0.651 (0.483–0.819)	0.614 (0.449–0.778)	0.567 (0.385–0.749)	0.697 (0.529–0.865)
	Cutoff value ^1^	0.666	0.543	0.680	0.224	0.574	0.315	0.419	0.613
	Sensitivity	0.833	0.867	0.567	0.600	0.833	0.433	0.633	0.800
	Specificity	0.882	0.665	0.765	0.529	0.471	0.824	0.588	0.647
ROI_6_	AUC (95% Confidence interval)	0.840 (0.718–0.962)	0.775 (0.598–0.951)	0.637 (0.466–0.808)	0.629 (0.470–0.789)	0.572 (0.396–0.4747)	0.627 (0.468–0.786)	0.522 (0.349–0.694)	0.776 (0.640–0.913)
	Cutoff value ^1^	0.711	0.581	0.597	0.245	0.637	0.317	0.397	0.598
	Sensitivity	0.700	0.833	0.767	0.533	0.533	0.422	0.733	0.833
	Specificity	0.882	0.824	0.529	0.765	0.647	1.000	0.412	0.606

AUC = area under the curve, ADC = apparent diffusion coefficient, MK = mean kurtosis, ODI = orientation dispersion index, fiso = isotropic volume fraction, ficvf = intracellular volume fraction, v-csf = free water fraction, v-extra = extra-axonal volume fraction, v-intra = intra-axonal volume fraction. ^1^ Cutoff values of the univariate binary logistic regressions are shown.

**Table 3:** Diagnostic performance to differentiate astrocytomas, IDH mutant and oligodendrogliomas, IDH mutant all diffusion kurtosis imaging (DKI), neurite orientation dispersion and density imaging (NODDI) and diffusion microstructure imaging (DMI) parameters in all regions of interest (ROI).

		ADC	MK	ODI	fiso	ficvf	v-csf	v-Extra	v-Intra
ROI_1_	AUC (95% Confidence interval)	0.615 (0.398–0.832)	0.604 (0.381–0.827)	0.647 (0.431–0.863)	0.631 (0.413–0.849)	0.529 (0.294–0.764)	0.652 (0.443–0.862)	0.572 (0.339–0.805)	0.668 (0.443–0.894)
	Cutoff value ^1^	0.558	0.549	0.301	0.732	0.594	0.694	0.586	0.472
	Sensitivity	0.824	0.824	0.529	0.353	0.941	0.529	0.824	1.000
	Specificity	0.455	0.455	0.727	0.909	0.273	0.818	0.364	0.455
ROI_2_	AUC (95% Confidence interval)	0.802 (0.621–0.984)	0.802 (0.619–0.986)	0.625 (0.398–0.853)	0.636 (0.421–0.852)	0.652 (0.421–0.884)	0.759 (0.576–0.942)	0.540 (0.308–0.772)	0.749 (0.548–0.950)
	Cutoff value ^1^	0.514	0.808	0.300	0.558	0.584	0.468	0.570	0.439
	Sensitivity	0.882	0.824	0.824	0.824	0.765	0.941	0.882	0.941
	Specificity	0.455	0.818	0.455	0.455	0.555	0.455	0.273	0.555
ROI_3_	AUC (95% Confidence interval)	0.818 (0.649–0.988)	0.749 (0.547–0.950)	0.658 (0.440–0.876)	0.599 (0.374–0.824)	0.599 (0.364–0.834)	0.674 (0.475–0.890)	0.460 (0.229–0.691)	0.647 (0.423–0.871)
	Cutoff value ^1^	0.606	0.909	0.290	0.587	0.590	0.621	0.611	0.467
	Sensitivity	0.824	0.765	0.882	0.824	0.706	0.765	0.412	0.941
	Specificity	0.727	0.636	0.455	0.364	0.636	0.627	0.364	0.455
ROI_4_	AUC (95% Confidence interval)	0.738 (0.544–0.932)	0.674 (0.454–0.893)	0.674 (0.472–0.876)	0.604 (0.382–0.826)	0.460 (0.231–0.707)	0.631 (0.402–0.860)	0.516 (0.287–0.745)	0.540 (0.305–0.775)
	Cutoff value^1^	0.535	0.907	0.339	0.584	0.617	0.514	0.636	0.579
	Sensitivity	0.824	0.765	0.529	0.765	0.059	0.941	0.294	0.765
	Specificity	0.636	0.627	0.436	0.545	0.636	0.455	0.909	0.455
ROI_5_	AUC (95% Confidence interval)	0.781 (0.600–0.961)	0.658 (0.442–0.874)	0.620 (0.411–0.830)	0.647 (0.428–0.866)	0.481 (0.240–0.723)	0.652 (0.438–0.867)	0.551 (0.327–0.774)	0.561 (0.331–0.792)
	Cutoff value ^1^	0.489	0.995	0.338	0.512	0.585	0.631	0.547	0.488
	Sensitivity	0.882	0.588	0.588	0.941	1.000	0.588	0.765	1–000
	Specificity	0.636	0.818	0.627	0.364	0.273	0.536	0.273	0.273
ROI_6_	AUC (95% Confidence interval)	0.797 (0.620–0.974)	0.631 (0.408–0.854)	0.508 (0.290–0.726)	0.556 (0.317–0.795)	0.503 (0.265–0.740)	0.567 (0.325–0.809)	0.561 (0.322–0.801)	0.548 (0.317–0.779)
	Cutoff value ^1^	0.523	0.935	0.340	0.518	0.499	0.531	0.585	0.521
	Sensitivity	0.882	0.765	0.529	1.000	1.000	1.000	0.882	1.000
	Specificity	0.727	0.545	0.182	0.273	0.273	0.364	0.364	0.182

AUC = area under the curve, ADC = apparent diffusion coefficient, MK = mean kurtosis, ODI = orientation dispersion index, fiso = isotropic volume fraction, ficvf = intracellular volume fraction, v-csf = free water fraction, v-extra = extra-axonal volume fraction, v-intra = intra-axonal volume fraction. ^1^ Cutoff values of the univariate binary logistic regressions are shown.

## Data Availability

In order to safeguard the confidentiality of the participants, the data pertaining to this study are currently withheld from public access. The data can be shared upon request.
